# Bone Injury and Repair Trigger Central and Peripheral NPY Neuronal Pathways

**DOI:** 10.1371/journal.pone.0165465

**Published:** 2016-11-01

**Authors:** Cecília J. Alves, Inês S. Alencastre, Estrela Neto, João Ribas, Sofia Ferreira, Daniel M. Vasconcelos, Daniela M. Sousa, Teresa Summavielle, Meriem Lamghari

**Affiliations:** 1 Instituto de Investigação e Inovação em Saúde (i3S), Universidade do Porto, Porto, Portugal; 2 Instituto de Engenharia Biomédica (INEB), Universidade do Porto, Porto, Portugal; 3 Faculdade de Medicina, Universidade do Porto (FMUP), Porto, Portugal; 4 Instituto Ciências Biomédicas Abel Salazar (ICBAS), Universidade de Porto, Porto, Portugal; 5 Instituto de Biologia Molecular e Celular (IBMC), Universidade do Porto, Porto, Portugal; 6 Escola Superior de Tecnologia da Saúde do Porto, Instituto Politécnico do Porto, Porto, Portugal; Universite de Lyon, FRANCE

## Abstract

Bone repair is a specialized type of wound repair controlled by complex multi-factorial events. The nervous system is recognized as one of the key regulators of bone mass, thereby suggesting a role for neuronal pathways in bone homeostasis. However, in the context of bone injury and repair, little is known on the interplay between the nervous system and bone. Here, we addressed the neuropeptide Y (NPY) neuronal arm during the initial stages of bone repair encompassing the inflammatory response and ossification phases in femoral-defect mouse model. Spatial and temporal analysis of transcriptional and protein levels of NPY and its receptors, Y1R and Y2R, reported to be involved in bone homeostasis, was performed in bone, dorsal root ganglia (DRG) and hypothalamus after femoral injury. The results showed that NPY system activity is increased in a time- and space-dependent manner during bone repair. Y1R expression was trigged in both bone and DRG throughout the inflammatory phase, while a Y2R response was restricted to the hypothalamus and at a later stage, during the ossification step. Our results provide new insights into the involvement of NPY neuronal pathways in bone repair.

## Introduction

Neuropeptide Y (NPY) is a 36 amino-acid peptide widely distributed in the central and peripheral nervous system, which has been shown to contribute to a broad range of physiological processes including control of feeding behaviour, energy homeostasis, vascular and immune function, pain and stress copping [1–3]. In the last decade, NPY was also identified as a powerful regulator of bone mass through both central- and peripheral-mediated pathways [4, 5]. Of the five known NPY receptors (Y1R, Y2R, Y4R, Y5R and Y6R), Y1R and Y2R were reported to be involved in bone homeostasis [4–6]. The induced overexpression of hypothalamic NPY in NPY-deficient mice resulted in a significant reduction in bone mass [7] and, in the same line, the conditional deletion of Y2R in the hypothalamus promoted an increase of bone mass [4]. These results point towards a central NPY-mediated control of bone mass through the Y2R in the hypothalamus. Several reports revealed that bone is innervated by NPY-positive fibers from the sympathetic nervous system [8, 9]. Moreover, NPY is also expressed by non-neuronal cells found in the bone microenvironment such as bone cells (osteoblasts and osteocytes), bone marrow cells and endothelial cells [10, 11]. So far, only the Y1R was demonstrated to be expressed in bone, namely by osteoblasts and bone marrow cells [12–14], further supporting a functional role for the NPY system in bone homeostasis. In fact, germline and osteoblast-specific Y1R deletion was shown to promote an increase in bone mass [5, 15], and the same effect was obtained after the pharmacological blockage of this receptor [16].

Evidence for the involvement of NPY signalling when bone homeostasis is severely compromised has also been provided. The gonadectomy-induced bone loss was overcome in germline or hypothalamic-specific deletion of the Y2R in both male and female mice [17]. In a fracture scenario, the number of NPY-positive fibers in the callus was increased [18] and the deletion of Y1R was shown to cause a delay in the repair process [19].

Current data on the sensory nervous system show that NPY is expressed in response to nerve injury [20–22], being involved in anti-hyperalgesic effects [23], while no detectable levels of NPY protein are found under basal conditions (i.e. non-injury) in the dorsal root ganglia (DRG), where the cell bodies of sensory neurons are located. NPY is also suggested to have a role in the regulation of angiogenesis and in angiogenesis-dependent tissue repair. Germline Y2R knockout mice exhibit a delay in skin wound healing and the blockage of NPY-induced angiogenesis [24].

However, how the interplay between the NPY neuronal pathways and bone injury occurs remains to be clarified.

Bone repair is characterized by an orchestrated sequence of events, in which multiple factors interact in a precise temporal and spatial cascade, resulting in the restoration of normal bone anatomy and function [25, 26]. Typically in bone repair, four overlapping phases are considered: (1) the hematoma and inflammatory response, (2) the soft callus formation, (3) the hard callus formation and finally (4) the bone remodelling phase [26]. Of these four steps, the inflammation stage was shown to be critical for the success of bone repair. Inflammatory cells attracted to the bone lesion area are involved in the recruitment of further inflammatory and mesenchymal cells, stimulating angiogenesis and enhancing extracellular matrix synthesis [27–29], with the consequent promotion of bone repair. NPY, acting through its receptors in immune cells, such as leucocytes, antigen-presenting cells and macrophages, is an important modulator of the immune system activity [30–32]. This suggests a putative role of NPY in bone repair through the regulation of the inflammatory reaction. Still, whether and how the NPY system activity is targeted during the initial steps of bone repair is presently not known.

In this study we show that the central and peripheral NPY pathways are triggered by bone injury and during repair in a time- and space-dependent manner. NPY-Y1R response is implicated in both bone and DRG during the inflammatory stage of bone repair while Y2R response is constrained to the hypothalamus and occurs later on, during the initial stages of bone tissue formation.

## Results

### Non-critical bone defect mouse model: animal health and bone healing progression

The animal health and bone healing progression was evaluated in C57BL/6 mice with a non-critical defect in the femur diaphysis.

**Fig 1:**
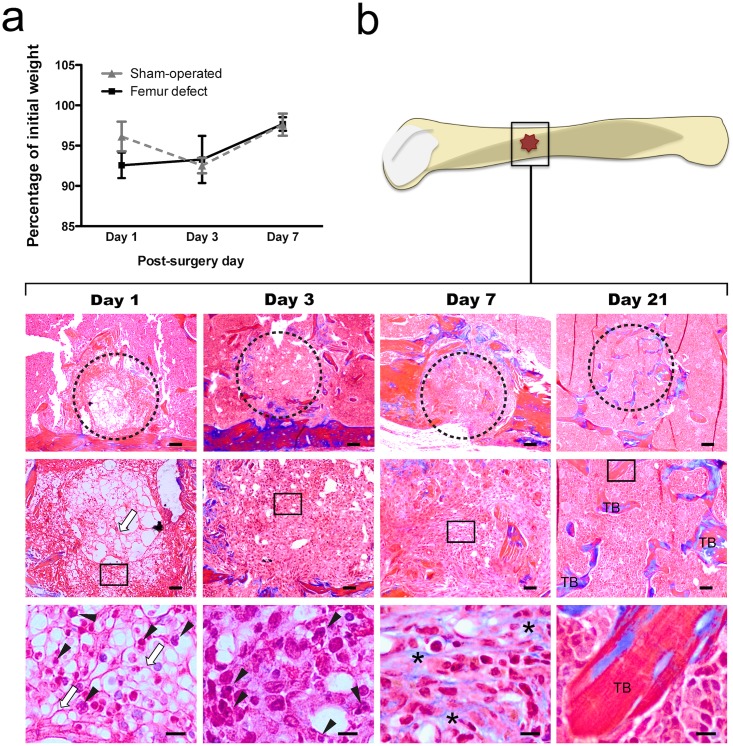
Body weight variation and bone healing progression in femur-defect vs sham-operated mice. The body weight variation (a) and bone healing progression (b) were evaluated in the femur-defect and sham-operated animals at day 1, 3 and 7 post-surgery. (a) Femur-defect and sham-operated mice presented no differences in body weight evolution. (b) The progression of bone healing after non-critical femur defect was evaluated in histological sections of femur stained with Masson’s trichrome. Day 1 was characterized by the presence of a high number of polimorphonucleated cells (black arrowhead) in a dense network of fibers (white arrow) in the defect (dashed circle). At day 3, the defect was filled by the granulation tissue, and at day 7 high amounts of collagen (*) were observed in the extracellular matrix. Bone trabeculae (TB) were observed in the defect area at day 21 post-defect. Upper line, Scale bar = 100 μm; Middle line, Scale bar = 50 μm; Lower line, Scale bar = 10 μm.

Animal mortality was not observed during surgical procedure or recovery period. Mice were able to move after recovering from anaesthesia and no differences in body weight were detected after surgery between femur-defect and sham-operated groups (Fig 1a). During the recovery period, no macroscopic signals of an exacerbated inflammatory reaction, such as redness and swelling, were identified in the skin wound.

The progress of bone healing was also monitored by histological analysis at day 1, 3, 7 and 21 post-surgery (Fig 1b). These time points encompass the i) acute inflammation (day 1); ii) (non-acute) inflammation and cellular proliferation (day 3) and iii) resolved inflammation state and beginning of ossification (day 7). Day 21 was evaluated as a control for the success of the repair process. At day 1, the defect was filled by a dense network of fibers with abundant entrapped erythrocytes and unfiltered cells with lobed nuclei ((polimorphonuclear cells (PMLs)). At day 3 post-defect the network of fibrin fibers was replaced by new connective tissue (granulation tissue). At this point, the PMLs and erythrocytes numbers were largely reduced in comparison to day 1. Day 7 was characterized by an intense deposition of extracellular matrix rich in collagen and no PMLs nor erythrocytes were detected. Sections from 21 days post-surgery showed that new trabeculae were formed at the edge and centre of the defect.

### NPY system within bone microenvironment is responsive to bone defect

To assess whether the local NPY system within bone is impacted during the early phases of bone repair, the mRNA expression levels of NPY and Y1R were assessed at day 1, 3 and 7 post-surgery in a segment of bone around the defect area and compared to the expression in a similar fragment from sham-operated mice.

**Fig 2:**
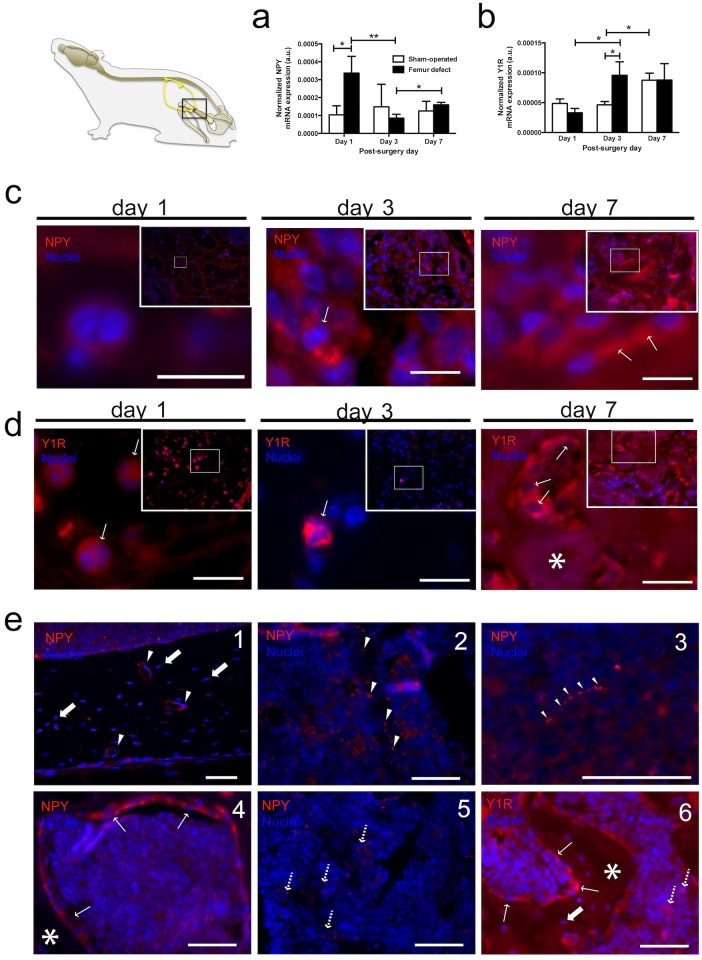
NPY system within bone microenvironment is responsive to bone defect. NPY (a) and Y1R (b) mRNA expression levels were assessed at days 1, 3 and 7 post-surgery in femurs from femur-defect and sham-operated mice. (a) Femur-defect mice displayed 2.5-fold higher NPY mRNA expression levels at day 1 and (b) 2-fold higher Y1R mRNA expression levels at day 3 as compared to sham-operated animals. In (a) and (b) each column represents the mean + SEM, for 8 animals per group. *p<0.050; ** *p*<0.010. NPY (c) and Y1R (d) immunoreactivity were assessed in femoral histological sections from femur-defect and sham-operated mice. Within the defect, polymorphonuclear cells stained for NPY (c) and Y1R (d) were observed at day 1 post-defect. NPY-positive and Y1R-positive cells were also observed within the granulation tissue at day 3. At day 7 a high number of NPY- and Y1R-positive osteoblasts (c and d, respectively) were observed surrounding the newly formed bone. Panel (e) shows NPY and Y1R immunoreactivity in the areas adjacent to the defect. NPY-positive nerve fibers were observed alongside blood vessels in the bone (1; white arrowhead) and in the bone marrow (2; white arrowhead), and also scattered in the bone marrow (3; white arrowhead); NPY and Y1R immunoreactivity was also observed for osteoblasts (4 and 6, respectively; thin white arrow), osteocytes (1 and 6, respectively; thick white arrow) and bone marrow cells (5 and 6, respectively; dashed arrow). * indicates bone tissue. Panel (c) and (d)- scale bar = 10 μm; Panel (e)- scale bar = 50 μm.

The NPY mRNA expression was 2.5-fold higher in the femur-defect mice at day 1 post-surgery as compared to the sham-operated mice (p = 0.011) (Fig 2a). In sham-operated animals, the NPY mRNA levels were stable throughout the assessment period.

Concerning Y1R, femur-defect animals showed a 2-fold increase of the mRNA expression levels from day 1 to day 3 post-defect (p = 0.020) that was maintained to day 7 (Fig 2b). In sham-operated animals, Y1R mRNA was unchanged from day 1 to day 3 and increased to levels similar to the ones found for femur-defect animals at day 7 post-surgery (p = 0.020) (Fig 2b). The expression of Y2R mRNA in bone was not detected.

NPY and Y1R protein expression was also analysed by immunohistochemistry in femur sections from femur-defect and sham-operated mice. Results showed that, in the defect area, NPY and Y1R were expressed by the same type of cells (Fig 2c and 2d). At day 1, a high number of NPY-positive and Y1R-positive PMLs were observed entrapped in the network of fibrin fibers (Fig 2c and 2d). These cells were mostly absent at day 3 and at this time point only a few cells within the granulation tissue were NPY or Y1R-positive (Fig 2c and 2d). At day 7 numerous NPY-positive and Y1R-positive cells were found within the borders of new bone tissue (identified as osteoblasts) in the defect area (Fig 2c and 2d). At all time points, no NPY-positive nerve fibers were found in the defect area. The analysis of the areas adjacent to the defect revealed the presence of numerous NPY-positive fibers localized mostly alongside blood vessels in bone tissue and bone marrow (Fig 2-e1 and 2-e2), and also scattered within the bone marrow (Fig 2-e3). NPY and Y1R staining was also observed in osteoblasts, osteocytes and in bone marrow cells (Fig 2-e4, 2-e5 and 2-e6). This pattern of NPY distribution was also observed in the femurs from sham-operated mice.

### Femur defect impacts NPY pathway in the sensory nervous system

The involvement of the NPY pathway within the sensory nervous system after bone injury was assessed by analysis of NPY, Y1R and Y2R mRNA expression in the L2-L5 DRG at day 1, 3, and 7 post-surgery in femur-defect and sham-operated animals.

**Fig 3:**
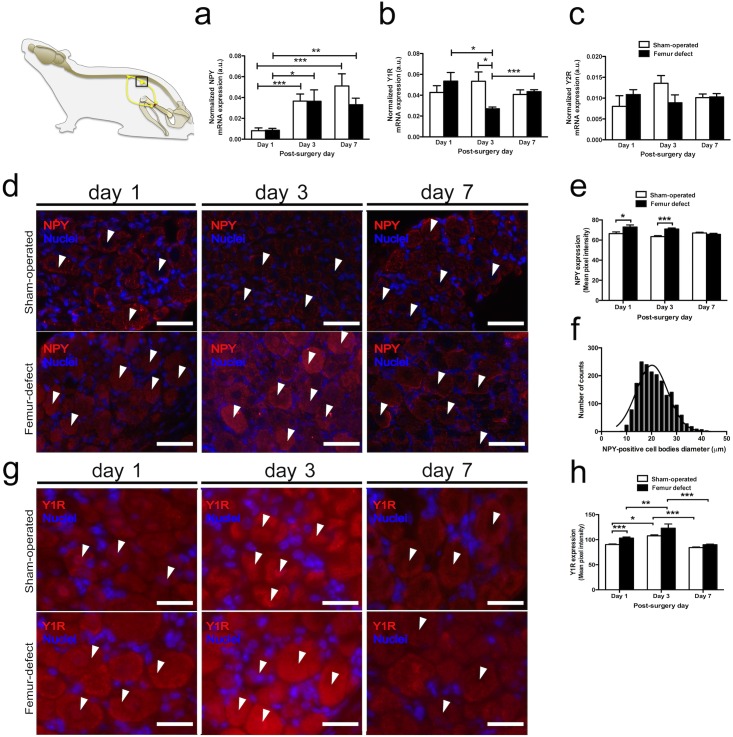
Impact of bone defect on the NPY neuronal pathways in the sensory nervous system. The mRNA expression levels of NPY (a), Y1R (b) and Y2R (c) in the DRG from femur-defect and sham-operated mice were analysed at day 1, 3 and 7 post-surgery. (a) NPY mRNA expression presented a 4-fold increase from day 1 to day 3 post-surgery in both femur-defect and sham-operated mice, which was sustained at day 7. (b) At day 3, femur-defect mice displayed a decrease in the Y1R mRNA expression to half of the expression levels found in sham-operated animals. (c) Y2R mRNA expression showed no differences between femur-defect and sham-operated mice. In (a), (b) and (c) each column represents the mean + SEM, for 8 animals per group. *p<0.050; ** *p*<0.010; *** *p*<0.005. Panel (c) and (d) show NPY and Y1R immunoreactivity in DRG sections, respectively. The quantification of NPY and Y1R staining intensity was performed and is shown in (e) and (h), respectively. Femur-defect mice displayed higher NPY immunoreactivity in the DRG at day 1 and 3 as compared to sham-operated (d and e). The Y1R immunoreactivity was also higher in femur-defect mice at day 1, and increased between day 1 and day 3 in both femur-defect and sham-operated mice (g and h). Panel (d) scale bar = 50 μm; Panel (g) scale bar = 25 μm In (e) and (h) each column represents the mean + SEM, for 3 animals per group. *p<0.050; ** *p*<0.010; *** *p*<0.005.

NPY mRNA expression levels were found to be within the same order of magnitude in both the femur-defect and in the sham-operated mice, and presented a significant 4-fold increase from day 1 to day 3 post-surgery (femur-defect mice p = 0.012; sham-operated mice p = 0.003), that was sustained in day 7 (Fig 3a).

In sham-operated mice, Y1R mRNA expression was kept constant throughout the assessment period but, in contrast, at day 3 post-injury, femur-defect mice displayed a significant reduction on Y1R mRNA expression levels to half of the ones found in sham-operated mice (p = 0.011) (Fig 3b). At day 1 and day 7, no differences were observed between the two experimental groups regarding the Y1R mRNA expression.

Y2R mRNA expression levels were kept stable and no differences between the two experimental groups were found (Fig 3c).

The differences regarding NPY and Y1R mRNA expression were further investigated by immunohistochemistry analysis in the DRG sections. Results confirmed the expression of NPY and Y1R in the DRG of both femur-defect and sham-operated mice (Fig 3d and 3g). Still, in the femur-defect animals NPY was found to be expressed at higher levels at days 1 and 3 post-surgery (p = 0.02 and p = 0.0008, respectively) (Fig 3d and 3e). In sham-operated mice no alterations in NPY expression were detected. NPY was found to be expressed by cells with cell bodies diameter ranging from 7 to 42 μm (Fig 3f). Concerning the Y1R, the expression was found to be higher in the femur-defect animals at day 1 (p = 0.0001), and to increase from day 1 to day 3 post-surgery in both sham-operated and femur-defect animals (p = 0.0159 and p = 0.0093, respectively) (Fig 3g and 3h).

### The hypothalamic NPY neuronal pathway is targeted during bone repair

The contribution of the hypothalamic NPY system during bone injury was assessed by analysis of NPY, Y1R and Y2R mRNA expression in the hypothalamus at day 1, 3, and 7 post-surgery in femur-defect and sham-operated mice.

**Fig 4:**
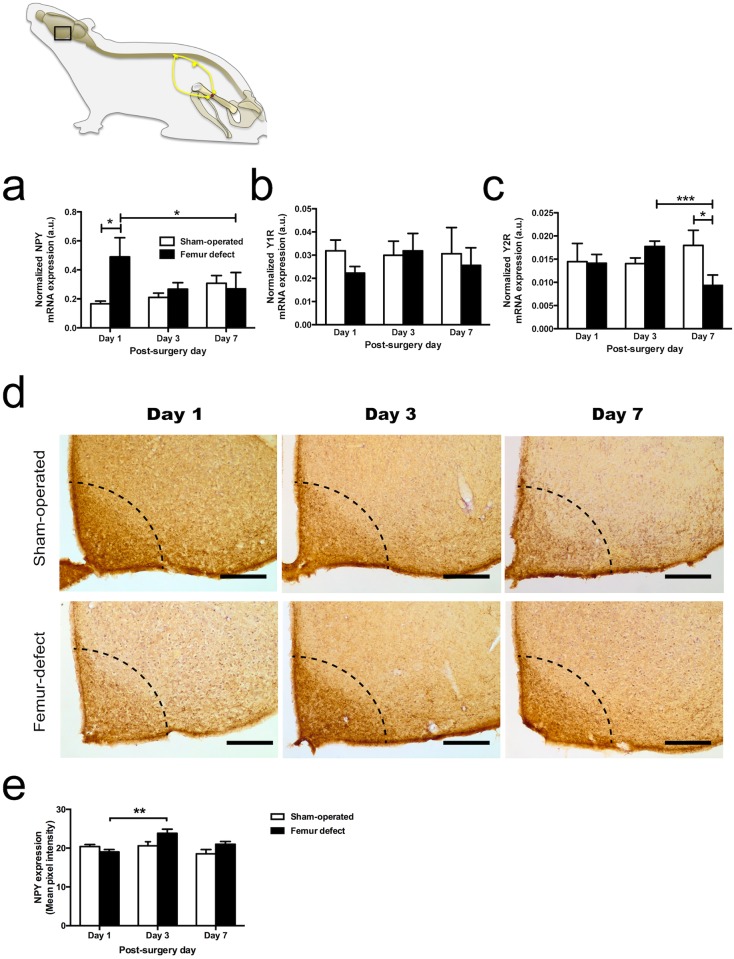
The hypothalamic NPY neuronal pathway is targeted during bone repair. The mRNA expression levels of NPY (a), Y1R (b) and Y2R (c) in the hypothalamus of femur-defect and sham-operated mice were assessed at days 1, 3 and 7 post-surgery. (a) Femur-defect mice presented a 2.5-fold higher NPY mRNA expression at day 1 and (c) a 2-fold lower Y2R mRNA expression at day 7, as compared to sham-operated mice. (b) No differences were observed in the Y1R mRNA expression between femur-defect and sham-operated mice. In (a), (b) and (c) each column represents the mean + SEM, for 8 animals per group. *p<0.050, *** *p*<0.005. Panel (d) shows NPY immunoreactivity in the hypothalamic section and in (e) the data from the quantitative analysis of the NPY staining intensity is provided. Femur-defect mice presented an increase in the NPY staining intensity in the arcuate nucleus from day 1 to day 3 post-surgery. Scale bar = 200 μm. In (e) each column represents the mean + SEM, for 3 animals per group. ** *p*<0.010;.

The comparison between femur-defect and sham-operated animals showed significant differences in the hypothalamic NPY and Y2R mRNA levels: in femur-defect mice the expression of hypothalamic NPY mRNA was 2.5-fold higher at day 1 post-surgery while hypothalamic Y2R mRNA was found to be reduced to half at day 7 (p = 0.010 and p = 0.028, respectively) (Fig 4a and 4c). The hypothalamic Y1R mRNA remained unaltered in both groups during the assessment period (Fig 4b).

The differences found in the NPY mRNA expression levels were then further explored by immunohistochemistry analysis in hypothalamic brain sections. Results showed an increase in NPY staining in the arcuate nucleus of the femur-defect mice between day 1 and 3 post-surgery (p = 0.0095) (Fig 4d and 4e).

Although several attempts were performed, immunohistochemistry analysis of Y2R failed due to the lack of an efficient commercially available antibody. Y1R immunohistochemistry was not performed in consequence of the lack of differences observed during hypothalamic mRNA expression analysis.

## Discussion

The present study shows that the central and peripheral NPY pathways are targeted during the initial stages of bone repair, pointing to an increase in the NPY system activity in response to bone injury. The results suggest a putative role for NPY on the biological events occurring early in bone repair.

As indicated by the histological analyses performed in bone sections, and in accordance with published data [33], bone injury triggers a sequence of inflammatory events characterized by the presence of numerous immune cells in the defect area (acute inflammation stage—day 1). Following, inflammation is reduced and starts to be resolved, as indicated by the clear reduction in the number of inflammatory cells (PMLs) (reduced inflammation stage-day 3). By day 7, PMLs are no longer detected and an intense deposition of extracellular matrix rich in collagen is observed, suggesting that inflammation is ended and ossification is initiated (ossification stage-day 7).

Several studies have shown that the NPY system is an important regulator of the immune activity, namely through Y1R. In fact, Y1R is known to modulate the activity of macrophages, dendritic and T cells [34, 35], and also to regulate the PMLs trafficking [36]. Within the bone microenvironment, NPY signalling through the Y1R is also known to decrease osteoblast activity [37].

In our study, we observe an increase of NPY and Y1R transcription in the defect area during the inflammatory phase (increased NPY at day 1, increased Y1R at day 3). As no similar effect is observed in sham-operated animals, these results indicate that the NPY system is being specifically responsive to a bone lesion and may play a role in the regulation of the resulting inflammatory response. Moreover, immunohistochemistry analysis revealed that during this stage, PMLs recruited to the defect area were the main cell type expressing both NPY and Y1R, suggesting an autocrine effect of NPY and further suggesting a role of this system in the regulation of the inflammatory stage. Upon the resolution of inflammation (day 7) there is a drastic change in the cell type population present at the defect area. PMLs are no longer present and osteoblastic cells, expressing both NPY and Y1R, are observed, suggesting that the local NPY-Y1R system may be also involved in the ossification step of bone repair.

At all time points, no NPY-positive nerve fibers were observed in the defect area indicating that NPY is being produced by non-neuronal sources, namely the above mentioned PMLs and osteoblasts. Additionally, we have also observed the lack of TH-positive fibers (sympathetic nerve fibers marker) from bone defect area (S1 Fig). In fact, a repulsion of sympathetic nerve fibbers (responsible for NPY release) from inflamed tissue has been reported as a mean to allow the formation of an inflammation-area [38], which is crucial to the normal repair process.

In the sensory nervous system, NPY is reported to be expressed only in response to a tissue or nerve injury [20–22], where it plays a nociceptive modulatory function by acting on Y1R and Y2R [39]. In agreement, our results show an increase in the DRG NPY transcription in both femur-defect and sham-operated mice, suggesting a broad response of the NPY system to tissue injury and not a bone defect specific reaction. However, immunohistochemistry analysis showed an increase of NPY protein expression during the inflammatory stages (day 1 and day 3) of bone repair, which was not found in the sham-operated animals. This result suggests that a distinct NPY post-transcriptional regulation, potentially related with the higher extension of tissue damage in the mice with femur defect, may be occurring. In fact, a positive correlation between the NPY upregulation and the extension of lesion was previously showed in nerve constriction [20], and the post-transcriptional regulation of NPY has also been suggested [40]. In particular, inflammatory signalling was shown to rapidly regulate the protein transcription [41, 42] and is a putative underlying cause for the differences found between the mRNA and protein expression. Y1R protein expression in DRG was also found to be upregulated during the inflammatory response to a bone defect (at day 1), and also to increase during this period in the sham-operated control group. Y1R are located in small to medium sized sensory neurons [43, 44], and are frequently found co-expressed with CGRP [44, 45]. Evidence supports an inhibitory function of the Y1R in nociceptive behaviour achieved, at least in part, due to the inhibition of neurosecretion [45, 46]. Therefore, changes in the NPY-Y1R system in the DRG may be associated with an anti-hyperalgesic effect.

NPY was also demonstrated to be involved in the regulation of bone metabolism through signalling on the hypothalamus, in a process involving the Y2R [4]. Our results show that also the hypothalamic NPY system responds during the inflammatory stages of bone repair, as indicated by an increase in mRNA expression of NPY at day 1 and an increase in the protein expression of NPY from day 1 to day 3 post-surgery.

It has been reported that hypothalamic NPY is responsive to metabolic- and stress-related processes [2, 3]. In our study, however, as both groups were subjected to the same surgical stress and no differences in body weight evolution were found, such underlying causes for hypothalamic NPY response can be excluded. Instead, the increased expression of NPY after femur defect is potentially related with the involvement of the hypothalamic NPY system in the nociceptive modulation during inflammation. In fact, NPY acting through the Y1R in the arcuate nucleus was demonstrated to exert an antinociceptive effect during peripheral inflammation in the rat [47]. Thus, the high expression of NPY may represent an attempt to counteract the increased nociception associated with the inflammatory reaction to bone defect.

In contrast, the hypothalamic Y2R was not responsive during the inflammatory stages of bone repair, but was downregulated at day 7, a time point that matches the beginning of the ossification process. The Y2R is primarily localized presynaptically, acting as an autoreceptor and inhibiting the expression of NPY [48, 49], and the deletion of the Y2R in the hypothalamus was shown to result in an increase of the bone mass formation [4]. In consequence, by reducing the NPY signalling response in the hypothalamus, the observed decrease in the Y2R transcription at the ossification step may be a putative response toward the formation of bone mass.

Altogether, these results show that the NPY system is responsive to bone injury in a time- and space-dependent manner. During the inflammatory stage of bone repair, NPY pathways were targeted in bone, DRG and hypothalamus. The peripheral regulation of the inflammatory stage may be Y1R-mediated, since the Y1R expression was trigged in both bone and DRG. Upon the resolution of inflammation and beginning of ossification only the central NPY-Y2R pathway was impacted.

## Methods

### Animals

All animal procedures were approved by the IBMC/INEB ethics committee and by the Portuguese Agency for Animal Welfare (Direção-Geral de Alimentação e Veterinária), in compliance with the European Community Council Directive of September 22, 2010 (2010/63/UE). Experiments were conducted by FELASA C graded researchers and all efforts were made to minimize the number of animals used and their suffering.

Three months old C57BL/6 male mice were provided by the Animal house of IBMC/INEB Associated Laboratory. Mice were kept under controlled conditions (20–22°C, 60% humidity and 12:12 hour light/dark cycle). Water and appropriate food were supplied ad libitum. Animals were randomized into the different experimental groups (n = 8).

### Surgical procedure

Animals were subjected to anaesthesia by intraperitoneal administration of 50 mg/kg ketamine (Clorketam 1000, Vétoquinol S.A., Lure, France) and 10mg/kg medetomidine (DEXDOMITOR, Orion Corporation, Espoo, Finland). Following, a 1 cm skin incision was performed along the upper leg, and muscle was carefully retracted between gluteus superficialis along the fascia latae biceps femoris exposing the femur. A cylindrical defect was performed in the femur diaphysis by using a 23 G needle. On the surgery day analgesia was provided by the administration of 1μg/g body weight of butorfanol (Butador, Richer Pharma AG, Wels, Austria). The sham-operated mice underwent the same surgical procedure described above but the femurs were left intact.

### Tissue sampling

#### mRNA expression analysis

At day 1, 3 and 7 post-surgery mice were decapitated, the bone-defect area from the femur-defect animals was trimmed and an equivalent section was obtained from the femurs of sham-operated mice. DRG (L2-L5) were collected. Brains were collected and the hypothalamus was dissected according to the atlas by Paxinos and Watson [50]. All collected tissues were frozen in 2-methylbutane cooled over dry ice and stored at -70°C until further processing.

#### Histological and immunohistochemistry analyses

At day 1, 3 and 7 post-surgery, mice were deeply anesthetized with pentobarbital (EUTASIL, CEVA, Sante Animale, Portugal) and transcardially perfused with 4% paraformaldehyde in 0.2 M phosphate buffer (pH 7.4). Femurs, L2-L5 DRG and brain were removed and further processed for analysis.

Femurs were decalcified in 0.25M EDTA with 0.07% (w/v) glycerol (pH 7.4) for 4 weeks at 4°C on a constant shaker. Decalcified femurs and L2-L5 DRG were processed for paraffin embedding and serial 3 μm thick sections were cut in the microtome (RM2255, Leica Biosystems). For histological and immunostaining analyses sections were deparaffinised and dehydrated in a modified alcohol series.

Coronal sections of 20 μm were obtained from the collected brains on a freezing microtome (Thermo Scientific HM550, Thermo Fisher Scientific).

### Histological analysis

Serial femur sections, obtained as described above, were stained with Masson’s Trichrome and images were acquired with an Olympus CX31 light microscope equipped with a DP-25 camera (Imaging Software CellˆB, Olympus, Center Valley, PA, USA).

### Immunohistochemistry analyses

#### Bone

Serial femur sections from 3 animals in each group, obtained as described in Tissue sampling section, were placed in antigen retrieval buffer for 20 min at 98°C (TE Buffer, pH 9.0). Following, sections were blocked and permeabilized for 1h in blocking solution (10% NGF in PBS with 0.5% Triton-X-100) at room temperature, and then incubated overnight with rabbit anti-NPY (1:5000, Sigma-Aldrich, Germany) or anti-Y1R antibodies (1:500, ImmunoStar, Hudson, USA) at 4°C. For both staining experiments, signal detection was achieved using a goat anti-rabbit Alexa Fluor 568 antibody (1:200 in the NPY experiment and 1:1000 in the Y1R experiment, Molecular probes, Life Technologies) for 1h at RT. Sections were washed and mounted in a glass slide with VECTASHIELD Mounting Medium with DAPI (Vector Laboratories). Primary antibody specificity was controlled by analysis of samples were the antibody was replaced by blocking buffer (S2 Fig). Immunostaining images were acquired on an Axiovert 200 inverted microscope equipped with AxioVision 4.8 software (Carl Zeiss, Germany).

#### DRG

NPY immunohistochemistry on L2-L5 DRG was carried out using the M.O.M Basic Kit (Vector, Peterborough, UK) following the manufacturer's instructions. Sections from 3 animals in each group, obtained as described in Tissue sampling section, were incubated overnight at 4°C with the monoclonal NPY antibody in MOM buffer (kindly offered by E. Grouzmann, University Hospital, Lausanne, Switzerland) diluted 1:4000 in MOM buffer. For Y1R staining, sections were placed in antigen retrieval for 20 min at 98°C (TE Buffer, pH 9.0), blocked and permeabilized for 1h in blocking solution (10% NGF in PBS with 0.5% Triton-X-100) at room temperature, and then incubated overnight with rabbit anti-Y1R antibody (1:500ImmunoStar) at 4°C. In both experiments, a goat anti-rabbit Alexa Fluor 568 antibody (1:1000, Molecular probes, Life Technologies) was used for signal detection (incubation for 1h at RT. Sections were washed and mounted in a glass slide with VECTASHIELD Mounting Medium with DAPI (Vector Laboratories). Control sections where the primary antibody was replaced with blocking buffer were also assayed (S2 Fig). Images were captured on an Axiovert 200 inverted microscope equipped with AxioVision 4.8 software (Zeiss, Germany). NPY and Y1R staining intensity was quantified using a custom made program written in MATLAB (The MathWorks, version 2015a, Natick MA, USA). Fixed lower and upper bound values in the intensity levels were used to segment the regions of interest (ROI) by separating background from the foreground and remove eventual labelling artifacts. Mean statistics were calculated for the pixel intensities in the ROIs. The analysis of the NPY-positive cells area was performed using the NIH ImageJ software.

#### Hypothalamus

Immunoreactivity to NPY was carried out in free-floating sections using a M.O.M. Basic kit (Vector Laboratories, Peterborough, UK), following the instructions of the manufacturer. Sections from 3 animals in each group, obtained as described in Tissue sampling section, were incubated overnight at 4°C with the monoclonal NPY antibody (kindly offered by E. Grouzmann, University Hospital, Lausanne, Switzerland). Antigen visualization was made using streptavidin peroxidase and 3,3′–diaminobenzidine as a chromogen. The specificity of the immunoreactivity to the primary antibody was tested by omitting the primary antibody from the experimental procedure (S2 Fig). Images were captured on an Axiovert 200 inverted microscope equipped with AxioVision 4.8 software (Zeiss, Germany). The NPY staining intensity was quantified as described above for the immunohistochemistry experiments in DRG, using a custom made program written in MATLAB (The MathWorks, version 2015a, Natick MA, USA).

### Quantitative Reverse transcriptase PCR (RT-qPCR)

Total RNA was isolated from bone, DRG and hypothalamus using the TRIzol reagent (Invitrogen, Life Technology, Paisley, UK) according to the manufacturer’s specifications. RNA purity was estimated from the ratio of absorbance readings at 260 and 280 nm and only ratio between 1.8 e 2 were accepted. RNA quality was checked in agarose gel and RNA concentration was determined in a NanoDrop spectrophotometer (NanoDrop^™^ 1000 Spectrophotometer, Thermo Fisher Scientific, Wilmington, Delaware, USA NanoDrop). 1 μg of RNA from femur and hypothalamus, or 0.4 μg of RNA from DRG was reverse transcribed using the SuperScript^™^ First-Strand Synthesis System for RT-PCR (Invitrogen, Carlsbad, CA, USA). Reference genes were used as internal standards for normalization: Phosphoglycerate kinase 1 (Pgk1) was used for the DRG and hypothalamus; Cyclophylin (Cyclo) was used for the femur analysis. The RT-qPCR reactions, were performed on the iQ5 Multicolor Real-time PCR Detection System from Bio-Rad (Hercules, CA, USA), using the iQ^™^ SYBR^®^ Green Supermix (Bio-Rad Laboratories, Hercules, CA, USA) as follows: 1 cycle of 95°C for 15 min followed by 40 cycles of 95°C for 30 sec and 58°C for 30 sec (according to the primer annealing temperature), ending with a melting curve analysis to control for the amplification of a single gene product. Product fluorescence was detected at the end of the elongation cycle. Primer design was performed using the software Beacon Designer (Premier Biosoft International, Palo Alto, CA, USA). The primers used were as follows: NPY sense primer: 5’-gac act aca tca atc tca tca-3’; NPY antisense primer: 5’-aac aag ttt cat ttc cca tc-3’; Y1R sense primer: 5’-ctc gct ggt tct cat cgc tgt gga acg g-3’; Y1R antisense primer: 5’-gcg aat gta tat ctt gaa gta g-3’; Y2R sense primer: 5’-tgt tta gat aat ccc ttg ctt-3’; Y2R antisense primer: 5’-cac ttt cac ttc tac agt ttg-3’; Pgk1 sense primer: 5’-ggg aag cgg gtc gtg atg ag-3’; Pgk1 antisense primer: 5’-tgg gtt ggc aca ggc att ctc-3’; Cyclo sense primer: 5’-tgg tca acc cca ccg tgt tc-3’; Cyclo antisense primer: 5’-tag atg gac ctg ccg cca gt-3’. mRNA quantification was performed by comparative threshold cycle quantification (ΔC_t_ method) using Pgk1 or Cyclo as a reference genes.

### Statistic analyses

Weight evolution was analysed using ANOVA with repeated measures. For the RT-qPCR and Immunohistochemistry staining intensity data, since variables did not pass the homogeneity of variance test and did not follow normal distribution, analyses were performed using the non-parametric Kruskal-Wallis test followed by the Mann-Whitney U Test to assess statistical significant differences between femur-defect and sham-operated mice. Differences were considered at the significant level of p<0.05. All data are expressed as mean + SEM. Statistical analyses were performed using the software SPSS 14.0 (SPSS INC. Chicago, Illinois, USA).

## Supporting Information

S1 Fig Distribution of TH-positive fibers in the femur after bone defect. TH-positive nerve fibers were observed in the periosteum (b; white arrowhead), alongside blood vessels in bone (c; white arrowhead) and bone marrow (d; white arrowhead), and also scattered in bone marrow (e; white arrowhead). Negative control was obtained by the omission of the primary antibody (a). Scale bar = 50 μm.
(TIF)

S2 Fig Negative controls for the immunohistochemistry experiments. The omission of the primary antibody eliminated the staining of NPY in (a) bone, (b) DRG and (c) brain, and Y1R in (d) bone and (e) DRG. Scale bar = 50 μm.
(TIF)
