# Supplementary figures and images for: Bone Injury and Repair Trigger Central and Peripheral NPY Neuronal Pathways
Source: PLoS One. 2016 Nov 1;11(11):e0165465. doi: 10.1371/journal.pone.0165465 (PMC5089690; doi:10.1371/journal.pone.0165465)

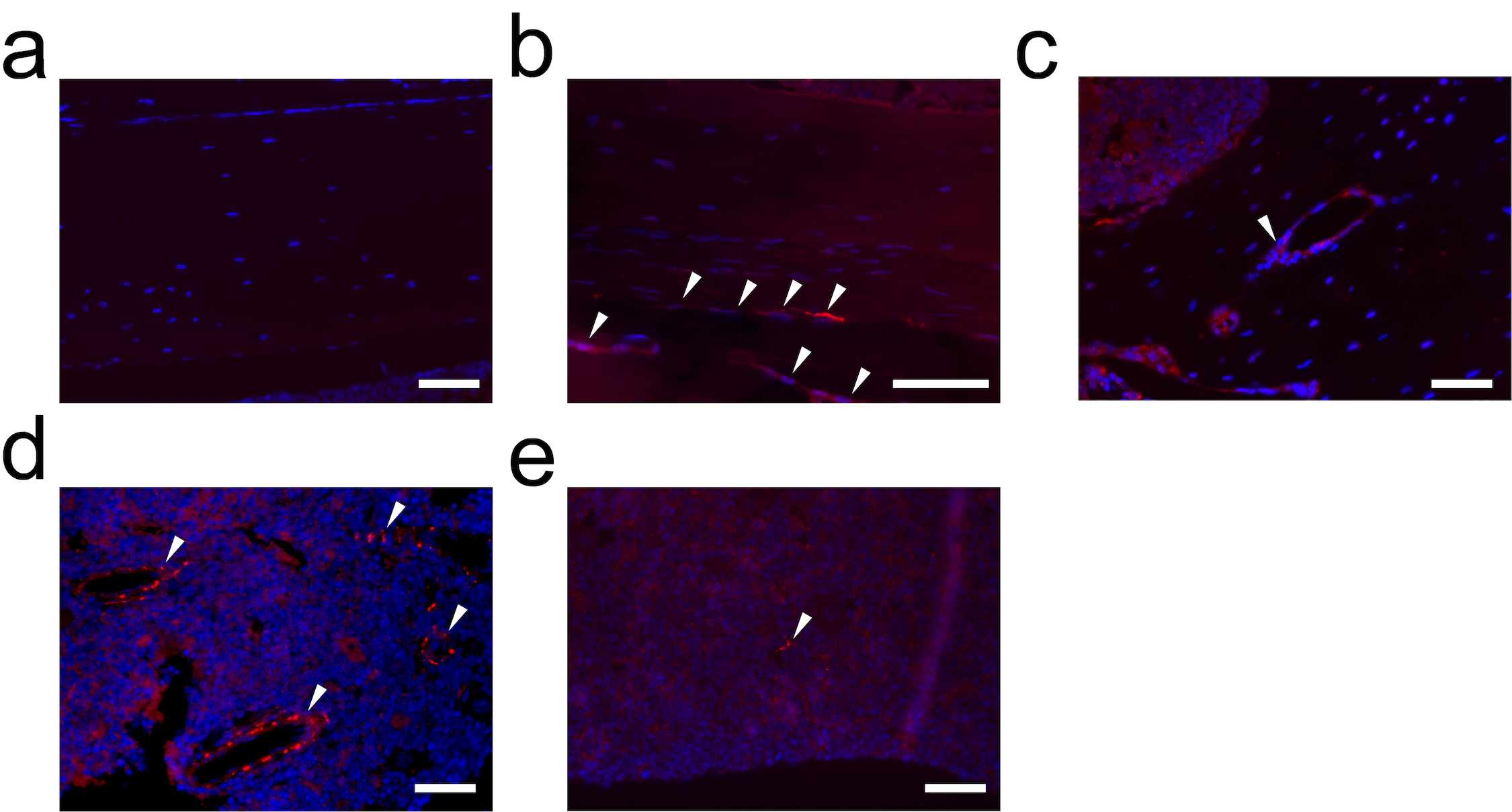

Supplement: S1 Fig — TH-positive nerve fibers were observed in the periosteum (b; white arrowhead), alongside blood vessels in bone (c; white arrowhead) and bone marrow (d; white arrowhead), and also scattered in bone marrow (e; white arrowhead). Negative control was obtained by the omission of the primary antibody (a). Scale bar = 50 μm. (TIF) [file pone.0165465.s001.tif]

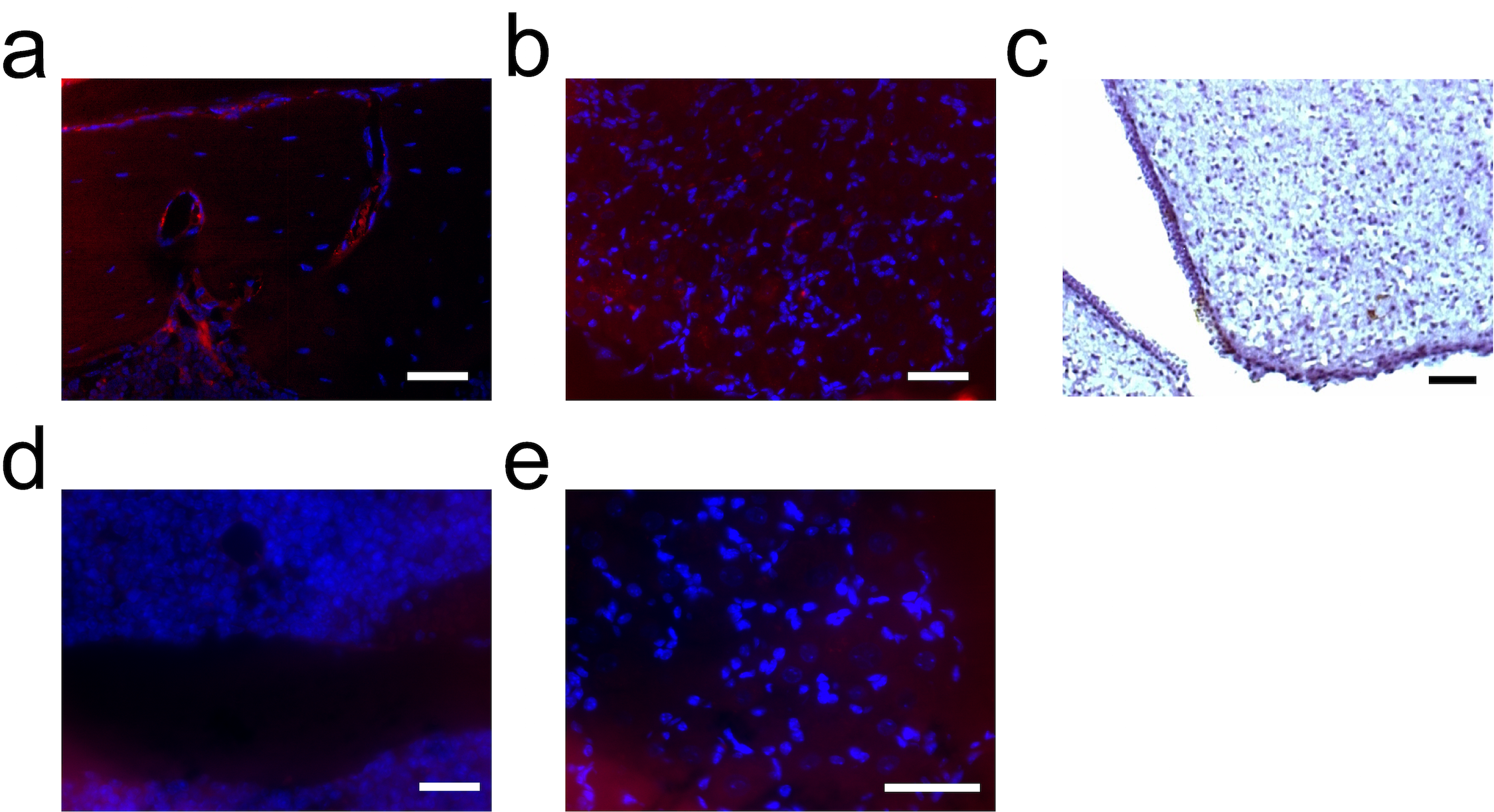

Supplement: S2 Fig — The omission of the primary antibody eliminated the staining of NPY in (a) bone, (b) DRG and (c) brain, and Y1R in (d) bone and (e) DRG. Scale bar = 50 μm. (TIF) [file pone.0165465.s002.tif]
